# Optically stimulated luminescence (OSL) dosimetry in irradiated alumina substrates from mobile phone resistors

**DOI:** 10.1007/s00411-017-0725-2

**Published:** 2017-12-18

**Authors:** Therése Geber-Bergstrand, Christian Bernhardsson, Maria Christiansson, Sören Mattsson, Christopher L. Rääf

**Affiliations:** Medical Radiation Physics, Department of Translational Medicine, Malmö, Skåne University Hospital Malmö, Lund University, 205 02 Malmö, Sweden

**Keywords:** OSL, Alumina substrates, Resistors, Fading, Emergency dosimetry, Retrospective dosimetry

## Abstract

In this study the dosimetric properties of alumina (Al_2_O_3_) substrates found in resistors retrieved from mobile phones were investigated. Measurements of the decline of optically stimulated luminescence (OSL) generated following exposure of these substrates to ionising radiation showed that 16% of the signal could still be detected after 2 years (735 days). Further, the magnitude of the regenerative dose (calibration dose; *D*_i_) had no impact on the accuracy of dose estimates. Therefore, it is recommended that the *D*_i_ be set as low as is practicable, so as to accelerate data retrieval. The critical dose, *D*_CL_, and dose limit of detection, *D*_DL_, taking into account the uncertainty in the dose–response relation as well as the uncertainty in the background signal, was estimated to be 7 and 13 mGy, respectively, 1 h after exposure. It is concluded that given the significant long-term component of fading, an absorbed dose of 0.5 Gy might still be detectable up to 6 years after the exposure. Thus, OSL from alumina substrates can be used for dosimetry for time periods far in excess of those previously thought.

## Introduction

In recent years, the field of emergency dosimetry has grown significantly, and continues to do so. An increasing awareness of the importance of this field has been driven by nuclear accidents and various other malevolent incidents that have occurred during the past few decades, such as the Fukushima Daiichi nuclear accident 2011 and the terrorist attacks in the US in September 11, 2001. Although radiation has not yet featured as part of a dedicated malicious attack, this possibility remains a consideration for which contingency planning is needed. A useful tool in the event of any nuclear incident is a robust and practical emergency dosimetry technique. Such a technique can help to distinguish between individuals that have been exposed to radiation and require medical follow-up vs. the un-exposed “worried well”.

There are numerous different techniques available for emergency dosimetry based on both biological and physical methods of assessment (Ainsbury et al. 2011). However, many of them still require additional research in order to be operable as emergency dose assessment methods following a large-scale emergency (McKeever and Sholom 2016). The optically stimulated luminescence (OSL) of electronic components [e.g. surface mounted resistors (SMRs) from mobile phones or other electronic devices] is one of the several potential methods that can be used. The dosimetric properties of SMRs originate from the alumina (Al_2_O_3_) substrate from which they are made. SMRs have proven to be promising in intra-laboratory comparisons and appear to satisfactorily be able to differentiate between individuals that have received whole-body absorbed doses above or below a threshold of 2 Gy (Bassinet et al. 2014). For immediate medical care, the 2 Gy cut-off for a whole, or a near-total body absorbed dose, has been suggested to be appropriate in large-scale emergencies (JIWG 2005; Bailiff et al. 2016). With this in mind, any emergency dosimetry method used must be able to determine this limit with good margins in terms of measurement accuracy.

OSL of electronic components offers a relatively fast method of dose assessment (results within hours), with a minimum detectable absorbed dose (MDD) under laboratory conditions, using read-out techniques currently available, estimated to be in the order of tens of mGy (e.g. Inrig et al. 2008; Kouroukla et al. 2014). One of the characteristics of this technique is that the OSL signal declines over time, a process generally referred to as fading. Normally, fading is considered to be a non-desirable property given that the signal is eventually lost. However, a more complete understanding of the properties of fading could, of itself, allow relevant information to be retrieved. This information might even be of use in scenarios where no OSL signal remains, with the proviso that no other factors that could have eroded the signal were involved (such as optical bleaching). In other words, if no OSL signal can be observed after a given period of time since the exposure, then it can be deduced that the individual either received no dose at all, or received a calculable maximum dose. A well-characterised fading function could also be used to determine the time of exposure by making two consecutive measurements, separated by a known time interval, and then correlating the decline in the OSL signal with the fading function.

The present study aimed to further investigate the fading properties of SMR alumina substrates from electronic components in contemporary mobile phones, in order to extract as much information as possible from each OSL measurement. To enable the translation from OSL signal to estimated dose, each sample was given a so-called regenerative dose or calibration dose (termed *D*_i_). For the purposes of emergency dosimetry, the *D*_i_ is ordinarily set to 5 Gy (e.g. Bassinet et al. 2014). The OSL signal arising from this *D*_i_ is then correlated to the OSL signal provided by an unknown dose (termed *D*_u_). Currently, it is thought that the relative magnitudes of *D*_i_ and *D*_u_ might affect the accuracy of dose estimation (i.e. comparable *D*_i_ and *D*_u_ values might provide a better dose estimate), a possibility that is also explored in this study.

The MDD is considered to be one of the parameters that defines the quality and utility of a specific retrospective dosimetry technique. Typically, the MDD is calculated as being three times the standard deviation of the signal originating from background or unexposed samples. However, this may not be sufficiently accurate given the uncertainty in the transition from OSL signal to dose (Geber-Bergstrand et al. 2012). Consequently, an alternative estimate is also provided in the present study.

## Materials and methods

**Fig. 1:**
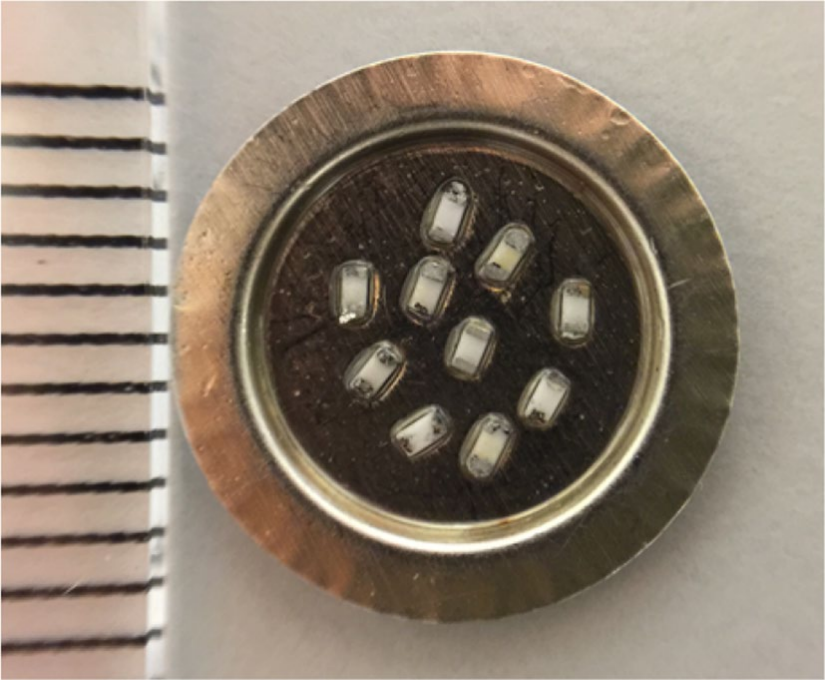
Alumina substrates (ten/cup) attached by oil spray to a stainless steel measuring cup. Each tick mark on the scale (at left) denotes 1 mm

The dose–response and fading properties of alumina substrates from SMRs removed from mobile phones were assessed. The particular resistors that possess dosimetric properties have a black cover with a white alumina substrate beneath; these resistors come in various sizes; the ones used in this study had the dimensions 1.0 × 0.5 × 0.35 mm^3^ (Fig. 1). A varying number of these resistors can be found in all modern mobile phones. The resistors used here have been extracted from various models and brands of mobile phones collected from the recycling bin at a local mobile phone vendor. The alumina substrates were extracted using a microscope, with the aid of a small screwdriver and tweezers. Then they were placed in stainless steel cups prepared with an oil spray (Rudolf medical, http://www.rudolf-med.com) to hold the alumina substrates in place. Irradiation was done with either a Risø TL/OSL-DA-15 reader equipped with a ^90^Sr/^90^Y source with a dose rate to resistors of 0.66 mGy/s, or a Risø TL/OSL-DA-20 reader with a ^90^Sr/^90^Y source with a dose rate to resistors of 130 mGy/s (DTU Nutech, Technical University of Denmark, Risø campus, Roskilde, Denmark). The details of these readers are described elsewhere (Bøtter-Jensen et al. 2000; http://www.nutech.dtu.dk). The weaker source has been calibrated directly against a ^60^Co source at IRSN (France); the dose rate of the stronger source was then calculated on the assumption that the ratio between the dose rates for resistors is the same as that (ratio) between the dose rates of the two sources in quartz, i.e. the standard material used to (dose) calibrate Risø equipment. Henceforth, the term “dose” refers to the dose rate to resistors from the weak ^90^Sr/^90^Y source, calibrated using the IRSN source.

Between irradiation and OSL read-out the alumina substrates were stored in either light-proof plastic boxes (formerly used to preserve photographic films), or were kept in the reader. When kept in the reader the aliquots were positioned in such a way that any leakage or scattered radiation from the incorporated source was negligible. Irradiated alumina substrates were handled under red light conditions, i.e. regular light bulbs covered with red plastic film (LEE filters, 106 primary red, http://www.leefilters.com).

All OSL read-outs were performed using the TL/OSL-DA-15 equipment, with the read-out parameters complying with protocols developed in the MULTIBIODOSE project (http://www.multibiodose.eu; Bassinet et al. 2014) termed “fast-mode” and “full-mode”. The fast-mode protocol simply consists of a read-out at room temperature for 30 s using continuous blue light stimulation (470 ± 30 nm, > 40 mW/cm^2^ at the sample position) delivering at 90% power. The full-mode protocol uses a pre-heat of 120 °C for 10 s (2 °C/s), followed by a read-out, after 5 s, at 100 °C (5 °C/s) for 30 s, using the same blue light stimulation delivering at 90% power (making the total time of the read-out approximately 115 s).

The net OSL signal was defined as the luminescence recorded by the photomultiplier tube (PMT) during the first 6 s of the read-out, subtracted by a background consisting of the luminescence recorded by the PMT during the subsequent 6 s [method as described by Bassinet et al. (2014)].

### The influence of the regenerative dose (*D*_i_)

The OSL-signals corresponding to the “unknown” doses (*D*_u_: 0.85 Gy; delivered by the weaker ^90^Sr/^90^Y source) were read-out using the full-mode protocol and then evaluated using *D*_i_ values of 0.5, 1, 2, and 5 Gy, given in that order. This same experiment was conducted using three aliquots (i.e. in triplicate), with ten alumina substrates per cup. The *D*_u_ was administered and its associated OSL signal retrieved before conducting each of the regenerative dose experiments (i.e. irradiation and OSL read-out). Individual estimates of *D*_u_ were derived by fitting a straight line with a zero intercept using an OSL vs. dose plot for the five different data combinations: for all *D*_i’s_ combined or to each of the four single *D*_i’s_. The estimated dose for each combination (taking the average of the three aliquots) was then compared to the *D*_u_ (Table 1); both signals (*D*_u_ and *D*_i_) were read at a time corresponding to 1 h after the midpoint of irradiation. Considering the very low sensitisation of alumina substrates (e.g. Pascu et al. 2013), the different *D*_i’s_ were delivered to the same samples after a bleach of 300 s following each OSL read-out (with illumination by blue LEDs) in order to quench the signal completely.

**Table 1:** Estimates of an “unknown” dose (*D*_u_) of 0.85 Gy (± 1 standard error of the mean), using either different single regenerative doses (*D*_i_) that range from 0.5 to 5 Gy, or all *D*_i’s_ simultaneously

	*D*_i_ (Gy)				
	0.5	1	2	5	All
Estimated dose (Gy)	0.90 ± 0.02	0.87 ± 0.01	0.89 ± 0.01	0.87 ± 0.01	0.87 ± 0.01
% of *D*_u_	106	102	105	102	102

The estimated doses represent the average of three different aliquots with alumina substrates. All irradiations were performed using a ^90^Sr/^90^Y source with a dose rate for resistors of 0.66 mGy/s

### Dose–response measurements

**Fig. 2:**
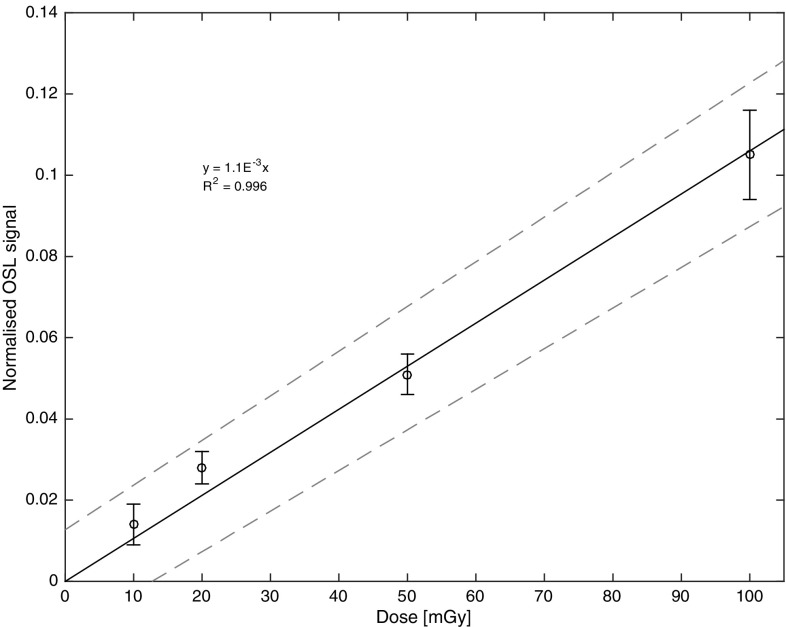
Dose–response curve of alumina substrates (± 1 standard error of the mean) at low doses, 1 h after exposure. Solid black line: regression function, as described in the figure; dashed lines: upper and lower 95% bound

The dose–response properties of alumina substrates have been studied on several occasions and can be described by a linear fit over a dose range from 0.01 to 150 Gy (e.g. Inrig et al. 2008; Bassinet et al. 2010; Pascu et al. 2013). In the present study, the focus was on the collection of dose–response data in the lower dose range, which was possible using the weaker ^90^Sr/^90^Y source. A multi-aliquot approach was used to construct the dose–response curve, i.e. for each administered dose, three aliquots with ten alumina substrates per cup were used. The doses administered were 10, 20, 50, and 100 mGy and a subsequent test dose of 1 Gy (for the purpose of inter-aliquot normalisation). The multiple-aliquot approach was chosen in order to obtain a more realistic uncertainty estimation of an individual measurement. All read-outs were performed both immediately after irradiation, as well as at a time point corresponding to 1 h after the midpoint of the irradiation using the full-mode protocol (Fig. 2).

**Fig. 3:**
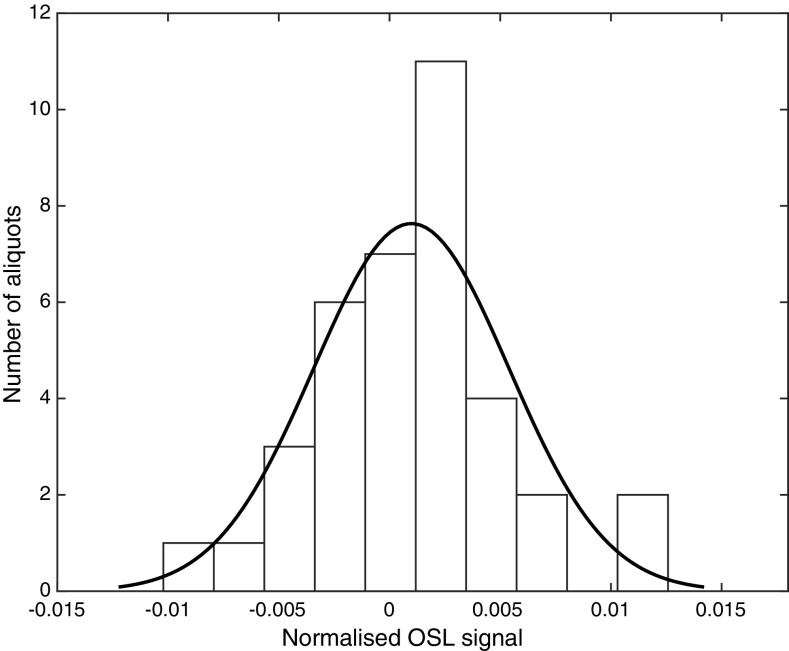
OSL signals of un-irradiated alumina substrates normalised to a test dose of 1 Gy, with a fitted normal distribution. A total of 37 aliquots were used, with ten alumina substrates per cup. The mean value was 0.001, and the standard deviation is 0.004

Although originally an idea from chemical metrology (Currie 1999, 2004), the terms critical dose, *D*_CL_, and dose limit of detection, *D*_DL_, have been used in the field of retrospective dosimetry (Ainsbury et al. 2017). In the present study, *D*_CL_ as well as *D*_DL_ is calculated while taking the uncertainty of both the dose–response fit and the zero-dose signals into account. By making a histogram of the normalised OSL signals that correspond to unexposed (zero-dose) samples (Fig. 3) read out with the fast-mode protocol, the average OSL signal for an unexposed sample and its corresponding standard deviation can be calculated. *D*_CL_ then describes the limit above which an observed signal exceeds the anticipated background with 95% probability (*α* = 0.05),

1 $${D_{{\text{CL}}}}=\frac{{\left\langle {{S_0}} \right\rangle }}{k}+1.645\left( {\sqrt {{{\left( {\frac{{{\sigma _{\text{S}}}}}{{\left\langle {{S_0}} \right\rangle }}} \right)}^2}+{{\left( {\frac{{{\sigma _{\text{k}}}}}{k}} \right)}^2}} \cdot \frac{{\left\langle {{S_0}} \right\rangle }}{k}} \right),$$

where $\left\langle {{S_0}} \right\rangle$ is the average of zero-dose samples and *σ*_S_ its corresponding standard deviation, *k* is the slope of the dose–response and *σ*_k_ its corresponding standard deviation. *D*_DL_ can in turn be estimated by replacing 1.645 in Eq. (1) with a factor 3 [roughly approximating 3.29·*σ*_B_ given for the Currie definition of detectable level for samples with well-known background (Currie 1968)].

### Exploration of the fading function

The following doses were used to study the fading effect: 1.7, 4.1, 8.3, and 41.3 Gy, all of which were obtained using the stronger ^90^Sr/^90^Y source. The OSL signal was then read at different time intervals post irradiation, commencing at 1 h (normalisation time point), and continuing for 2 years. OSL signals were not acquired for all time intervals and doses (see the “Results and discussion” section). For each time point, three aliquots (again with ten alumina substrates per cup) were measured. The OSL signal of each aliquot was normalised to the OSL signal from a 5 Gy test dose for the same aliquot. The OSL signal from the test dose was read out after 1 h in order to match the normalisation time point of 1 h established in the previous experiments. The full-mode protocol was used for all measurements except for the samples that were given a dose of 41.3 Gy. These samples were measured using the fast-mode protocol, in order to investigate the influence of a pre-heat on fading, as observed in earlier studies (Kouroukla et al. 2014). An assessment of the OSL signal decay in alumina substrates was performed using STATISTICA 10 software (TIBCO Software Inc, http://www.statistica.io). The half-lives of the different components of the OSL signal decay were extracted by means of non-linear regression. In order to obtain a proper fit of the long-term component, an initial regression with a mono-exponential decay model was used to fit data points after 1 year post exposure. In the second step of the regression, the obtained value of the half-life of the long-term component was set as a fixed parameter into a three-component exponential decay model. A subsequent regression of the model was then carried out using all the data points, also those within 1 year post exposure, thus extracting estimates of the half-lives for the other components.

## Results and discussion

### The influence of *D*_i_

When estimating an “unknown” dose, *D*_u_, by means of a regenerative (calibration) dose, *D*_i_, it was found that the magnitude of *D*_i_ had no impact on estimates of *D*_u_ (Table 1). Consequently, any dose of *D*_i_ could be used. Furthermore, the accuracy of the dose estimation was not improved if several different *D*_i_ doses were used. Given these findings, it is evident that neither the magnitude nor spread (i.e. varied doses) of *D*_i_ is a factor of importance in estimating *D*_u_. Consequently, the *D*_i_ can be relatively low (e.g. 1 Gy), in order to retrieve medical decision-making data (for the individual) as rapidly as possible.

### Dose–response measurements

The MDD is one of the parameters that can be used to evaluate the utility of any dosimetric technique. Traditionally, the MDD is defined as the dose corresponding to a signal equal to three times the standard deviation of unexposed (zero-dose) samples. When this approach is used, the uncertainty in the fit of the dose–response curve is not taken into account. The accuracy of the slope depends on the number of measurements included, allowing to generate a function that not only affects the accuracy with which the dose can be estimated, but also impacts the calculated MDD. A linear fit of the normalised OSL signals, read-out immediately after irradiation vs. the administered dose, is described by a function $y=0.0012 \pm 0.00004x$ (*R* ^2^ = 0.974). In practice, a sample from an emergency exposure would never be read out immediately. Therefore, also dose–response data 1 h after exposure were collected. In practise, even this time frame would likely be unrealistic in terms of an emergency exposure. However, this experiment just serves to illustrate the effects such short time periods can imply. In addition, the time point of 1 h post exposure may be usefully linked to the normalisation point of 1 h for the fading measurements (as discussed later). A linear fit of the OSL signal read-out 1 h from the midpoint of irradiation vs. the administered doses is described by a function$~y=0.001 \pm 0.00004x$ (*R* ^2^ = 0.996), see Fig. 2.

Table 2 shows the estimates of *D*_CL_ and *D*_DL_ immediately after exposure as well as 1 h after exposure. The traditional MDD is also shown for comparison. For immediate read-outs the *D*_CL_ and *D*_DL_ were 6 and 12 mGy, respectively. These values increased to 7 and 13 mGy, respectively, for read-outs 1 h after exposure. The traditionally calculated MDD of 11 mGy is in the same order of magnitude as the *D*_DL_.

**Table 2:** Dose limits for alumina substrates immediately after exposure and 1 h after exposure for normalised OSL signals (test dose of 1 Gy)

Immediately after exposure		1 h after exposure		Traditional MDD
*D*_CL_	*D*_DL_	*D*_CL_	*D*_DL_	3*σ*_zero-dose_
6	12	7	13	11

The critical dose (*D*_CL_) and the detection limit (*D*_DL_) accounts for both the uncertainty of the zero-dose readings and of the dose–response fit. The traditional calculation of the minimum detectable dose (MDD), corresponding to three times the standard deviation of the zero-dose readings, is also presented. All doses are given in mGy

### Fading measurements

The assessment of the signal decay in the alumina substrates shows that the fading process comprises more than one component. Three components were discerned; one was rapid, with a half-life (mean ± *σ*_mean estimate_) of 0.8 ± 0.1 days; one was intermediate, with a half-life of 22.3 ± 4.5 days; the final was slow, with a half-life of 790 ± 210 days.

**Fig. 4:**
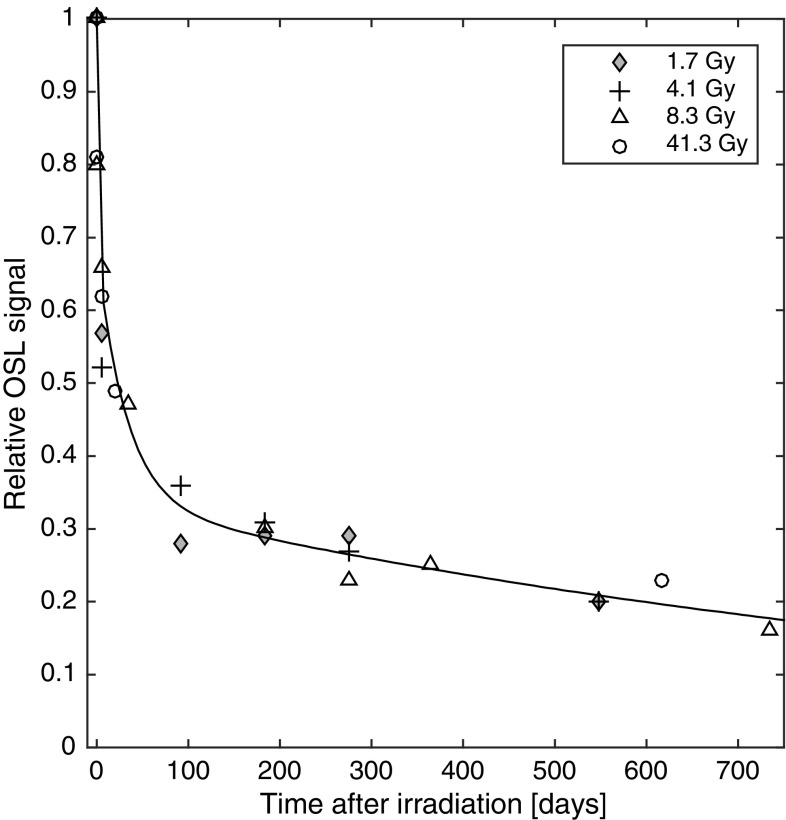
Fading of the OSL signal following doses of 1.7, 4.1, 8.3, and 41.3 Gy, for time intervals ranging from 1 h to 735 days. The corresponding three-component regression function is provided in Eq. (2)

The fading process observed appears to be independent of any given dose (Fig. 4). In addition, for the 41.3 Gy exposure, the fading appeared to be unaffected by the use of a preheat temperature of 120 °C. These data contrast with those reported by Kouroukla et al. (2014). The reason for this discrepancy remains unclear, but could reflect the different normalisation times used in the two analyses [1 h after irradiation in the present study vs. 60 s after irradiation for Kouroukla et al. (2014)]. The 1 h delay could potentially quench short-lived components in the alumina substrate that would otherwise have been registered shortly after irradiation. Nevertheless, the result reported here is very encouraging, as it supports the use of a universal fading correction for alumina substrates. This fading can be described by a function of the following form:

2 $$y=0.315{{\text{e}}^{ - 0.863t}}+0.348{{\text{e}}^{ - 0.031t}}+0.337{{\text{e}}^{ - 0.00087t}},$$

where *y* is the remaining OSL signal (percent), and *t* is the time elapsed since exposure (days).

Two years after exposure, 16% of the original signal remained. By using Eq. (2), as well as the value of the *D*_DL_ obtained after 1 h from the previous section, the time period for which a signal that corresponds to a certain dose can still be observed may be estimated. For example, a dose of 0.5 Gy may be measured up to 6 years after exposure, and a dose of 2 Gy may be seen up to 9 years after exposure. These values are provided with the caveat that the samples are not handled in any way that might degrade the signal, e.g. by being heated or exposed to light. An important note is that since the underlying mechanism of the fading behaviour is not yet completely known, the fading function could have a different appearance beyond that experimentally observed, and thus the calculated times for how long a signal could be seen might be subject to change in the future.

## Discussion

The results obtained in the present study suggest that OSL signals in alumina substrates corresponding to doses of interest in emergency dosimetry may be measured even several years after exposure. Until now, the longest post irradiation fade period that has been measured was 100 days. In the present study, it is now shown that 16% of the signal (relative to 1 h after exposure) can still be detected after 2 years (735 days). This indicates that OSL in alumina substrates can be used not only to retrieve dose estimates in the short-term (i.e. during the first few days after an incident), but also in the long-term. This finding is of relevance when considering the use of OSL in epidemiological studies of radiation effects. Furthermore, the fading of the OSL signal seems to be independent of the dose received by the alumina substrates, which is encouraging in terms of deriving a universal fading correction for dose estimation.

Interestingly, the magnitude of the regenerative dose (*D*_i_), delivered to a sample after obtaining the read-out of an unknown dose (*D*_u_), did not affect the accuracy of the dose estimation. Consequently, it is suggested that low regenerative doses of 1 Gy be used, in order to accelerate the read-out procedure, given that speed is of essence following an emergency exposure where the number of samples to be measured within a short period of time might be high.

In the present study, the MDD was calculated by using the OSL signals from unexposed (zero-dose) samples, and the *D*_CL_ and *D*_DL_ calculated by taking the standard deviations in both the zero-dose samples and the dose–response fit into account. The *D*_DL_ describes a dose corresponding to a signal for which, with a certainty of 95%, it can be said that a substrate has been exposed to radiation. The lowest values obtained was 6 mGy for *D*_CL_ and 12 mGy for *D*_DL_, which is in the same order of magnitude as previously reported (e.g. Inrig et al. 2008).

A limitation of the present study that was identified in hindsight is that for the fading experiments it would have been desirable to perform more measurements at approximately 80 days after exposure. As can be seen in Fig. 4, this time period represents a transition point for the fading process. Obviously, it would also be of value to have measurements that extend beyond 735 days. Since the fit of the dose–response data affects *D*_CL_ and *D*_DL_, further efforts should be made to expand this data set, i.e. collect more measurements for each dose and administer a greater number of doses in order to improve the accuracy of the regression slope.

## Conclusions

The fading of the OSL signal in alumina substrates from resistors in mobile phones was investigated for time periods following exposure to ionising radiation from 1 h to 2 years (735 days). The fading rate was found to be independent of the given dose and comprised three distinct components. After 2 years, 16% of the OSL signal measured 1 h after exposure remained. These data suggest that a dose of 0.5 Gy may be detected more than 6 years after the initial exposure. Thus, OSL in alumina substrates can be stably retrieved (with the caveats previously described) for time periods much longer than previously thought. In addition, the results support the use of a universal fading correction. This makes OSL on resistors an even more useful method for emergency and retrospective dosimetry than was considered until now. The size of the regenerative dose, *D*_i_, did not impact the estimated dose. Therefore, the *D*_i_ can be low in order to save time in retrieving medically relevant data. The *D*_CL_ and *D*_DL_ values reported here of 6–13 mGy are also consistent with previous studies, despite their different derivation. Future studies should investigate the long-term fading in more detail and for even longer time periods. It would be interesting to also look at other materials that have been rejected for use in OSL dosimetry because of their rapid initial fading as they might exhibit the same type of behaviour as resistors, i.e. a sharp decrease of the fading for extended periods of time.
